# An Unusual Presentation of Extracavitary Primary Effusion Lymphoma: Internal Jugular Vein Occlusion, Intractable Symptoms of Intracranial Hypertension, and Prolonged Remission after Highly Active Antiretroviral Therapy

**DOI:** 10.1155/2022/6046783

**Published:** 2022-04-23

**Authors:** Anindita Ghosh, Rodrick Chitaurirwa Zvavanjanja, Joshua Baalwa

**Affiliations:** ^1^Departments of Pathology, Laboratory Medicine, McGovern Medical School, University of Texas Health Sciences Center at Houston, Houston, Texas, USA; ^2^Diagnostic and Interventional Imaging, McGovern Medical School, University of Texas Health Sciences Center at Houston, Houston, Texas, USA

## Abstract

Primary involvement of the skeletal muscle by extracavitary primary effusion lymphoma (PEL) is an extremely rare phenomenon. We report an unusual case of PEL involving the jugulodigastric skeletal muscle without serous cavity involvement which resulted in complete occlusion of the ipsilateral proximal internal jugular vein, causing the patient to present with clinical features of intractable throbbing headache, photophobia, acute confusion state, sporadic syncopal attacks, and dyspnea without obvious palpable neck swellings. This led to an initial clinical suspicion, dedicated diagnostic workup, and empiric therapy for acute meningoencephalitis, severe atypical pneumonia, and acute pulmonary embolism. Owing to his refractory symptoms, exploratory CT imaging eventually revealed a heterogenous jugulodigastric mass, and finally, a pathologic diagnosis of extracavitary PEL was identified as the cause of his intracranial hypertension. The patient remains in remission 22 months after commencing a dolutegravir-based HAART regimen without any chemotherapeutic intervention.

## 1. Introduction

Primary effusion lymphoma (PEL) commonly presents as malignant effusions in the serous cavity. Extracavitary PEL without serous cavity involvement is rare and usually involves the lymph nodes, spleen, visceral organs, and bone marrow among other locations [1]. Similar to other non-Hodgkin lymphomas, primary soft tissue involvement is rare and is usually seen in advanced stages of nodal lymphoma as secondary involvement [2].

The jugulodigastric region is located below the posterior belly of the digastric and anterior to the proximal portion of the sternocleidomastoid muscle and provides space for major neck vessels coursing in and out of the cranium. Of the major neck vasculature, the internal jugular vein courses most closely to the proximal portion of the sternocleidomastoid muscle where it is susceptible to occlusion by jugulodigastric mass lesions. Occlusion of the internal jugular vein has been reported to cause significant morbidity including fatal intracranial hypertension [3].

The mechanism of pathogenesis of human herpesvirus type-8 (HHV8)-related PEL is multifaceted. HHV8 preferentially infects postgerminal center B cells and plasma cells and, upon integrating into their genome, produces multiple oncogenic viral transcripts which promote viral latency (latent-associated nuclear antigen), host cell proliferation (latent-associated nuclear antigen and viral cyclin), and resistance to apoptosis (viral FLICE inhibitory protein) [1]. It is therefore not surprising that antiviral therapy improves outcomes of patients with HHV8-associated primary effusion lymphoma.

Here, we report the first case of extracavitary PEL primarily involving the jugulodigastric skeletal muscle and causing symptoms consistent with intracranial hypertension. The patient remains in remission 22 months after commencing a dolutegravir-based HAART regimen without any chemotherapeutic intervention.

## 2. Case Report

A 31-year-old male presented with worsening dyspnea, excruciating throbbing headache, photophobia, and spotting in his vision for three days in November 2019. He reports that his symptoms were insidious during onset beginning approximately in July 2019. The dyspnea eventually became associated with a nonproductive cough and frequent episodes of fevers and chills, and in October of the same year, the symptoms became intolerable resulting in one week's hospitalization and empiric treatment for pneumocystis jiroveci pneumonia (PJP). He was discharged after feeling better on HAART and Bactrim regimens, for which he was poorly compliant. Three weeks later, he was again hospitalized with further worsening of dyspnea and throbbing headache, nausea, vomiting, diarrhea, and a syncopal episode. He did not require intubation but did require supplemental oxygen. After a week in hospital, he was discharged but at this time, he was off HAART. This recovery was short lived, and within three weeks from discharge, he presented again with rapid worsening of the same symptoms, as alluded to above in November 2019. His other past medical history included living with HIV/AIDS for 10 years, neurosyphilis, and hypertension. He is reported to have been compliant with HAART for only the first three years following HIV/AIDS diagnosis.

On examination, he was found to be tachypneic, mildly hypoxic, tachycardic, and hypotensive and his neck was supple with no obvious cervical lymphadenopathy. He had elevated serum lactate, but his LDH was within normal limits. His absolute CD4 count was 267 cells/*μ*L, and the HIV RNA viral load was 5.27 logs. Direct fluorescent antibody (DFA) studies on a bronchial alveolar lavage sample revealed neither *Pneumocystis jirovecii* organisms nor influenza A and B, parainfluenza 1, 2, and 3, adenovirus, nor respiratory syncytial viruses. Workup on sputum samples detected no acid-fast bacilli by Ziehl–Neelsen staining or culture. A QuantiFERON Gold test was ordered but was not performed by the reference laboratory due to sampling issues. EBV peripheral blood serology tests were ordered but not performed due to sample lipemia. A buffy coat smear exam for white blood cell intracellular microorganisms and urine histoplasma antigen were negative. Anerobic and aerobic blood cultures were negative. A chest X-ray showed retrocardiac atelectasis but no other definitive evidence of pneumonia or pulmonary edema. A chest CT for the pulmonary embolism protocol was also emergently performed and showed neither evidence of pulmonary embolism nor right heart strain but showed increased soft tissue density in the right lower neck with surrounding subcutaneous edema, noting that these findings are nonspecific and may represent contusion, represent neoplasm, or be related to a lymphoproliferative disorder or HIV. Nuclear medicine imaging also showed low probability of pulmonary embolism.

**Figure 1:**
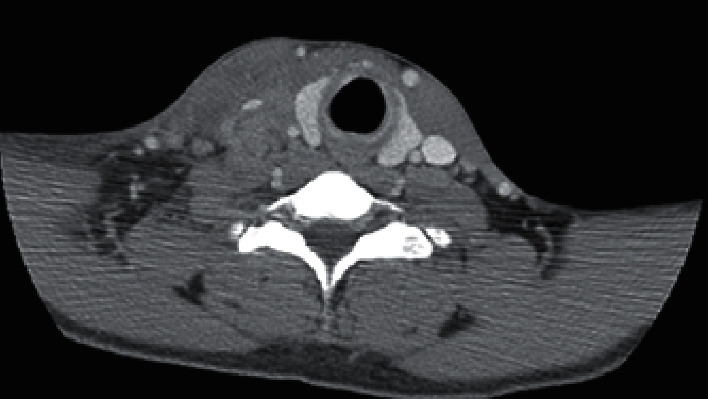
Pretreatment computed tomography of neck soft tissue with contrast, axial image shows right internal jugular vein compressed to a slit by a large heterogenous jugulo-digastric mass.

In view of his throbbing headache and photophobia, an urgent CT head followed by MRI head was performed to assess for space-occupying lesions and meningoencephalitis; however, both showed no acute abnormalities. A lumbar puncture was performed, and cerebrospinal fluid (CSF) analysis revealed a clear sample with a normal protein level and no pleocytosis. Additional CSF studies were negative for cryptococcal antigen, VDRL and treponemal antigen, aspergillus/blastomyces/histoplasma (both yeast phase/mycelial phase) antibodies, and herpes simplex virus and varicella zoster virus DNA by polymerase chain reaction. Due to findings of right neck edema and a dense lesion on the prior chest CT PE protocol, CT of the neck soft tissue was performed four days later due to further deterioration in presenting symptoms, and it revealed an enlarged and heterogenous right sternocleidomastoid muscle and a heterogenous right jugulodigastric confluent mass measuring approximately 7.4 cm in the greatest craniocaudal dimension. Transient compression upon the right internal jugular vein and complete occlusion of the proximal internal jugular vein by the heterogenous mass were also noted (Figure 1). An abdominal ultrasound scan was performed at the same time due to right upper quadrant discomfort, and it revealed no significant findings except for mild hepatosplenomegaly. CT abdomen was not performed.

**Figure 2:**
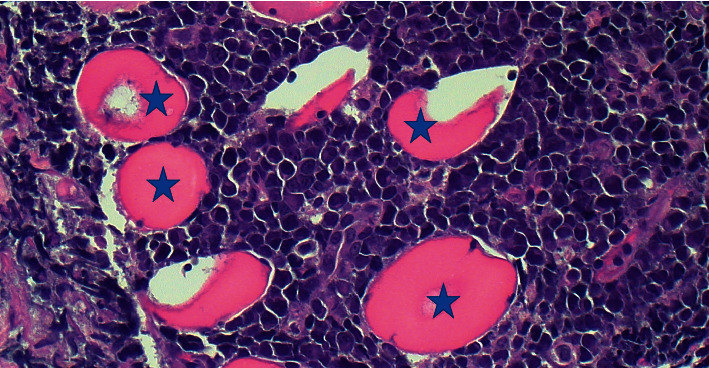
Primary effusion lymphoma involving skeletal muscle. Hematoxylin and Eosin stain shows skeletal muscle (blue asterixes) infiltrated and replaced by large neoplastic plasmacytoid cells. All images at 400X magnification.

**Figure 3:**
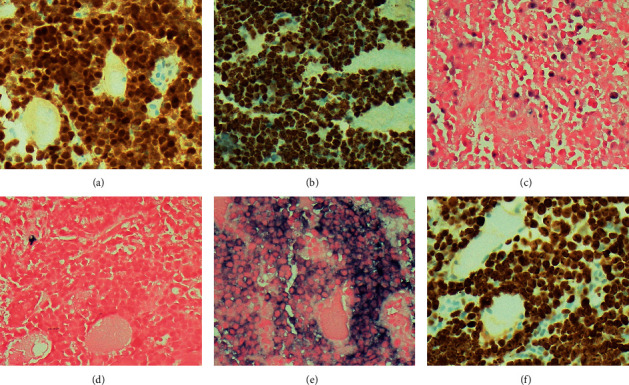
(a) Neoplastic cells diffusely positive for MUM-1. (b) HHV8 immunohistochemical stain is diffusely positive in neoplastic cells, while (c) a subset of neoplastic cells are positive for Epstein Barr virus encoded RNA by in situ hybridization staining. (d, e) The neoplastic cells show strong lambda light chain restriction (e) compared to kappa (d) light chain by in situ hybridization staining. (f) The neoplastic cells demonstrate a high proliferation index (<90%) as measured by Ki67 immunohistochemical stain. All images at 400X magnification.

Consequently, ultrasound-guided fine core needle biopsies of the 7 cm heterogenous mass stained by hematoxylin and eosin revealed tissue cores of the skeletal muscle infiltrated by sheets of large atypical plasmacytoid cells with predominantly vesicular chromatin, high nuclear to cytoplasmic ratio, variably conspicuous nucleoli, and scant to moderate cytoplasm (Figure 2). On immunohistochemical staining, the neoplastic cells were strongly and diffusely positive for MUM-1, EMA, and HHV8 and weakly for CD45, while in situ hybridization staining also revealed diffuse and strong staining for EBV-encoded RNA and strong lambda light chain restriction (Figure 3). They were negative for CD20, PAX5, CD79a, CD138, CD30, CD15, ALK-1, CD10, BCL6, and BCL2. Flow cytometric analysis of the same tissue biopsy revealed an abnormal B-cell population positive for CD45 (dim), CD38, CD138 (occasional cells), and cytoplasmic lambda light chain and negative for CD19, CD20, CD56, and cytoplasmic kappa light chain. In addition, fluorescent in situ hybridization studies on the core biopsies utilizing target DNA and control probes for MYC oncogene on the long arm of chromosome 8 (8q) revealed MYC amplification in 20 of 100 nuclei analyzed, further providing clonal support for a lymphoid neoplasm. Therefore, based on these morphological, immunophenotypical, and genetic findings, a final diagnosis of extracavitary PEL was made.

**Figure 4:**
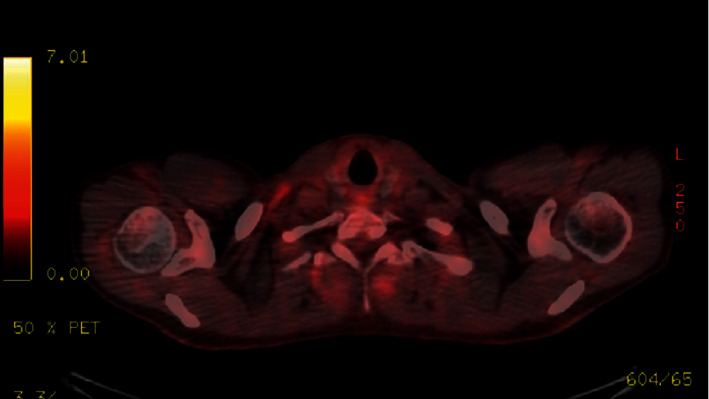
Immediate post treatment fused PET/CT. Seven weeks post initial head and neck CT. Approximate similar level demonstrating total resolution of the previous right heterogenous mass and right internal jugular compression. No fluorodeoxyglucose (FDG) avid lymph nodes seen.

**Figure 5:**
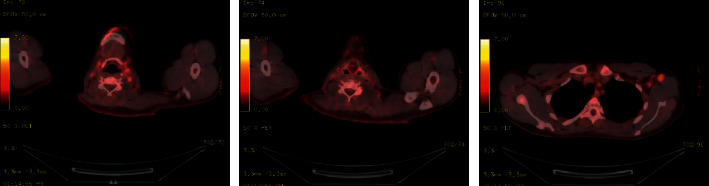
Second post treatment fused PET/CT demonstrating some FDG avidity in bilateral level 2 nodes and left axillary nodes which are reactive. The treated right jugulo-digastric mass not active or enlarged as demonstrated in Figure 1.

He was treated with oxygen therapy by nasal canula, intravenous fluids, broad-spectrum antibiotics, and prednisone empirically for PJP pneumonia, and his acute symptoms subsided. His HAART regimen was also changed from Genvoya (elvitegravir, cobicistat, emtricitabine, and tenofovir alafenamide) to dolutegravir and Descovy (emtricitabine and tenofovir alafenamide). Surprisingly, during the course of his HAART treatment, his right neck mass steadily resolved with eventual complete restoration of the right internal jugular vein patency as seen on consecutive staging PET scans (Figures 4 and 5). Additionally, both initial staging and restaging PET scans, approximately 7 and 42 weeks after diagnosis, revealed no FDG abnormal activity within the spleen nor within his abdomen or pelvic lymph nodes. After nearly two years of follow-up, the patient continues to experience sustained remission of his PEL without ever having received any chemotherapeutic intervention.

## 3. Discussion

The extracavitary variant of PEL is very rare and has unusual clinical presentations leading to significant diagnostic challenges. Its morphological and immunohistochemical findings are similar to classic PEL, although extracavitary varieties have a slightly better prognosis. Multiple previous case reports have shown the extracavitary form presenting as visceral, cutaneous, and intravascular tumors [4–7]. However, data on primary soft tissue involvement are sparse, yet their involvement could compromise function of adjacent vital neurovascular structures. This report thus adds information to the known possible protean de novo presentations of the extracavitary variant of PEL.

We found our case diagnostically challenging because it took multiple hospitalizations over at least four months before arriving at an unexpected diagnosis of hematological malignancy involving the nuchal skeletal muscle. Although our patient repeatedly presented with the same symptoms, progressively localizable to the head, a cause for symptoms remained an enigma after routine microbiological, biochemical, and imaging studies. Factors likely contributing to the diagnostic challenge for our case were masking of neck symptoms by excruciating headaches and the lack of obvious palpable neck lesions. In addition, heterogenous involvement of the jugulodigastric muscles by lymphoma, together with their deep location in the neck, likely precluded easy visibility of neck masses. Our diagnostic challenges were therefore not entirely unexpected and were further compounded by the fact that early diagnosis of nuchal lymphomas can be notoriously difficult [8].

Nuchal extraluminal vascular occlusion by lymphoma as a cause of cerebrovenous insufficiency including intracranial hypertension is very rare. In one study of 128 individuals, the commonest cause of cerebrovenous insufficiency due to extraluminal internal jugular vein compression was osseous bone lesions, with neck swollen lymph nodes accounting for only 1% [9]. Our patient manifested symptoms of intracranial hypertension secondary to extraluminal internal jugular vein compression as outlined in the modified Dandy criteria, with the exception that an increase in cerebrospinal fluid opening pressure was not measured. These symptoms included intractable headaches associated with photophobia and lack of a demonstrable CNS lesion on imaging. Due to these symptoms, our patient was worked up and empirically treated for meningoencephalitis, albeit with no sustained improvement.

Achieving prolonged remission of HIV-associated PEL with a HAART regimen alone without concurrent chemotherapy is extremely rare with less than ten published reports in the literature [10–13]. In these rare case reports, the benefit from HAART treatment is thought to be due to HHV8 viral burden control by antiretroviral-induced immune reconstitution and/or direct antiviral action of some antiretroviral medications on HHV8 [14]. The control of HHV8 infection results in elimination of viral-derived oncogenes and therefore tumor remission. Therefore, considering the rarity of primary effusion lymphoma and its overall poor treatment outcomes, we feel that continued reporting of rare clinical scenarios of treatment successes on a case-by-case basis is warranted to inform novel treatment strategies.

Notably, our patient developed primary effusion lymphoma while on an integrase inhibitor-based HAART regimen, Genvoya^R^. Following diagnosis of primary effusion lymphoma, his antiretroviral treatment was changed from Genvoya^R^ to a dolutegravir-based HAART regimen, and within four weeks, his neck mass resolved. This rapid remission coincided with a drop in the HIV viral load from 184,000 copies/mL to 7370 copies/mL although, paradoxically, his CD4 count instead dropped from 267 to 105 cells/microliter. He has remained in clinical remission without requiring chemotherapy for at least 22 months after switching to a dolutegravir-based HAART regimen, also an integrase inhibitor-based HAART regimen. Whether there might be differences in antitumoral efficacy among various integrase inhibitor-based HAART regimens against HHV8-associated PEL is still unknown and may be worthy exploring in larger case series. Differences in antitumoral efficacy have been reported for HHV8-associated Kaposi's sarcoma where protease inhibitors were shown to have better antitumoral efficacy compared to integrase and nonnucleoside reverse transcriptase inhibitors [15, 16]. Of note, Genvoya^R^ contains a boosted second-generation integrase inhibitor, while dolutegravir is a nonboosted third-generation integrase inhibitor. To our knowledge, our case is the second to report prolonged tumor remission on a dolutegravir-based HAART regimen [13].

## 4. Conclusion

In patients with unexplained symptoms of intracranial hypertension, it is worthwhile pursuing a differential diagnosis of nuchal lymphoma such as PEL as a possible cause.

## Data Availability

The data used to support the findings of this study are available from the corresponding author upon request.
